# DNA methyltransferase isoforms expression in the temporal lobe of epilepsy patients with a history of febrile seizures

**DOI:** 10.1186/s13148-019-0721-2

**Published:** 2019-08-19

**Authors:** Laurence de Nijs, Kyonghwan Choe, Hellen Steinbusch, Olaf E. M. G. Schijns, Jim Dings, Daniel L. A. van den Hove, Bart P. F. Rutten, Govert Hoogland

**Affiliations:** 1 School for Mental Health and Neuroscience (MHeNS), Department of Psychiatry and Neuropsychology, Faculty of Health, Medicine and Life Sciences, Maastricht University, Universiteitssingel 50, 6229 ER Maastricht, The Netherlands; 2 GIGA-Neurosciences, University of Liège, Liège, Belgium; 3 Department of Psychiatry, Psychosomatics and Psychotherapy, University of Würzburg, Würzburg, Germany; 4 Department of Neurosurgery, Maastricht University Medical Center, Maastricht, The Netherlands; 5 Academic Center for Epileptology (ACE), Maastricht University Medical Center, Maastricht, The Netherlands

**Keywords:** Febrile seizures, Temporal lobe epilepsy, Epigenetics, DNA methylation, DNA methyltransferases

## Abstract

**Background:**

Temporal lobe epilepsy (TLE) with hippocampal sclerosis (HS) is a common pharmaco-resistant epilepsy referred for adult epilepsy surgery. Though associated with prolonged febrile seizures (FS) in childhood, the neurobiological basis for this relationship is not fully understood and currently no preventive or curative therapies are available. DNA methylation, an epigenetic mechanism catalyzed by DNA methyltransferases (DNMTs), potentially plays a pivotal role in epileptogenesis associated with FS. In an attempt to start exploring this notion, the present cross-sectional pilot study investigated whether global DNA methylation levels (5-mC and 5-hmC markers) and DNMT isoforms (DNMT1, DNMT3a1, and DNMT3a2) expression would be different in hippocampal and neocortical tissues between controls and TLE patients with or without a history of FS.

**Results:**

We found that global DNA methylation levels and DNMT3a2 isoform expression were lower in the hippocampus for all TLE groups when compared to control patients, with a more significant decrease amongst the TLE groups with a history of FS. Interestingly, we showed that DNMT3a1 expression was severely diminished in the hippocampus of TLE patients with a history of FS in comparison with control and other TLE groups. In the neocortex, we found a higher expression of DNMT1 and DNMT3a1 as well as increased levels of global DNA methylation for all TLE patients compared to controls.

**Conclusion:**

Together, the findings of this descriptive cross-sectional pilot study demonstrated brain region-specific changes in DNMT1 and DNMT3a isoform expression as well as global DNA methylation levels in human TLE with or without a history of FS. They highlighted a specific implication of DNMT3a isoforms in TLE after FS. Therefore, longitudinal studies that aim at targeting DNMT3a isoforms to evaluate the potential causal relationship between FS and TLE or treatment of FS-induced epileptogenesis seem warranted.

**Electronic supplementary material:**

The online version of this article (10.1186/s13148-019-0721-2) contains supplementary material, which is available to authorized users.

## Introduction

Temporal lobe epilepsy (TLE) is the most common type of focal epilepsy in adulthood. TLE is drug-resistant in about one third of patients, necessitating surgical resection of the epileptogenic focus as a curative treatment option [1]. Drug-resistant TLE is frequently associated with hippocampal sclerosis (HS) as the major pathological entity. In general, HS is histopathologically characterized by astrogliosis and neuronal loss, most prominently in the cornu ammonis (CA) 1 and CA3 regions and in the dentate gyrus (DG) of the hippocampus. In addition, 50% of the patients with HS display various degrees of granule cell dispersion in the DG [2]. Typical febrile seizures (FS) are fever-triggered convulsions, affecting 2–14% of children between the age of 6 months and 5 years. FS have been linked to the development of subsequent TLE with HS in 30–40% of the cases [3]. Little is known about the pathophysiological mechanisms of TLE, especially after FS. In a well-characterized rodent model of FS, i.e., that of neonatal hyperthermia, FS have been shown to result in enhanced hippocampal excitability and consequently an increase in epileptic seizures [4]. Molecular and cellular changes have been demonstrated and correlated to the generation of this enhanced excitability (for review, see [5]). Identifying the mechanisms that regulate the transcription of genes that mediate these alterations may open new perspectives for the treatment of drug-resistant epilepsy or for the prevention of FS-induced epileptogenesis.

Epigenetic mechanisms, which refer to inherited and acquired changes in gene transcription that occurred without modification in the DNA sequence, have been proposed to play a prominent role in various neurological disorders, including epilepsy [6]. They are involved in multiple aspects of brain functions and associated behaviors, e.g., impacting on neuronal development, neuronal and synaptic plasticity, and memory [7]. DNA methylation, a key epigenetic modification implicated in the regulation of gene transcription, has been shown to be highly dynamic in the adult brain, e.g., in the hippocampus in response to neural activity [8], and it is thought to be crucial for brain homeostasis. DNA methylation is the covalent modification of cytosine residues on CpG di-nucleotides to form 5-methylcytosine (5-mC) that is catalyzed by DNA methyltransferases (DNMTs) [9]. 5-Hydroxymethylcytosine (5-hmC) is the oxidative variant of 5-mC and is generated during a reaction catalyzed by ten-eleven translocation enzymes [10]. The exact function of 5-hmC is still not fully elucidated, but evidence indicates that 5-hmC marks can regulate gene expression by driving DNA demethylation [11]. Moreover, 5-hmC has been shown to be highly abundant in the brain compared to other tissues [12].

Various types of DNMTs have been reported including DNMT1, DNMT3a, and DNMT3b. DNMT1, referred to as maintenance DNMT, conserves DNA methylation after every cellular DNA replication cycle, whereas DNMT3a and DNMT3b promote de novo DNA methylation. While more than 30 enzymatically active isoforms of DNMT3b exist [13], two isoforms of DNMT1 [14] and DNMT3a [15] have been described. For DNMT3a, these include the full-length isoform, known as DNMT3a1, and the shorter one, called DNMT3a2. DNMT3a2 lacks the 219 amino acids at the N-terminal part of DNMT3a1 and is transcribed from an alternative promoter in the sixth intron of the *DNMT3a* gene [15].

DNMTs have been shown to play an important role during brain development as inactivation of Dnmt1 in mouse leads to embryonic lethality [16] and DMNT3a/3b knockout mice die within few weeks postnatally [17]. The high expression of DNMT1, DNMT3a2, and DNMT3b in early stages of neurogenesis [15, 18, 19] decreases upon differentiation, while DNMT3a1 protein levels increase postnatally [19, 20]. Over the lifespan, the abundance of DNMT3a1 declines following synaptogenesis in neurons with very low levels during aging [19]. Remarkably, all the DNMT isoforms are still expressed in adult brain with DNMT1 and DNMT3a1 being more abundant than DNMT3b and DNMT3a2 [15, 18, 21, 22]. DNMT proteins were reported to be strongly expressed in neuronal as compared to glial cells [23].

In addition to their role during development, studies have demonstrated the functional importance of DNMTs and dynamic methylation in the adult brain, e.g., in learning and memory, synaptic plasticity, and behavior [24–28]. Recent evidences suggest that aberrant DNA methylation and expression of DNMTs may be causal or contributing factors in a variety of neurological disorders including epilepsies [26]. For example, preclinical data have shown altered DNA methylation patterns and DNMT activity in rodent models of status epilepticus and chronic TLE [29–31] as well as in human TLE [32–34]. In addition, the expression of DNMT1 and DNMT3a was shown to be increased in the neocortex of TLE patients [35].

Based on these findings, one could speculate that DNA methylation represents a crucial event triggered by FS, which leaves a permanent imprint on gene expression and physiological processes, thereby mediating epileptogenesis responsible for the development of TLE later in life. In order to start exploring this notion at a descriptive level, the present pilot study aimed to assess the expression of DNMT1 and DNMT3a isoforms and the level of global DNA (hydroxy)methylation in the pathogenesis of FS followed by TLE. We thereby tested the hypothesis that hippocampal and neocortical expression of DNMT1 and DNMT3a isoforms, as well as the levels of markers of global DNA methylation and hydroxymethylation (5-mC and 5-hmC, respectively), would be different between controls and TLE patients with or without a history of FS.

## Results

### Expression of DNMT1 in the neocortex and hippocampus of TLE patients

**Fig. 1:**
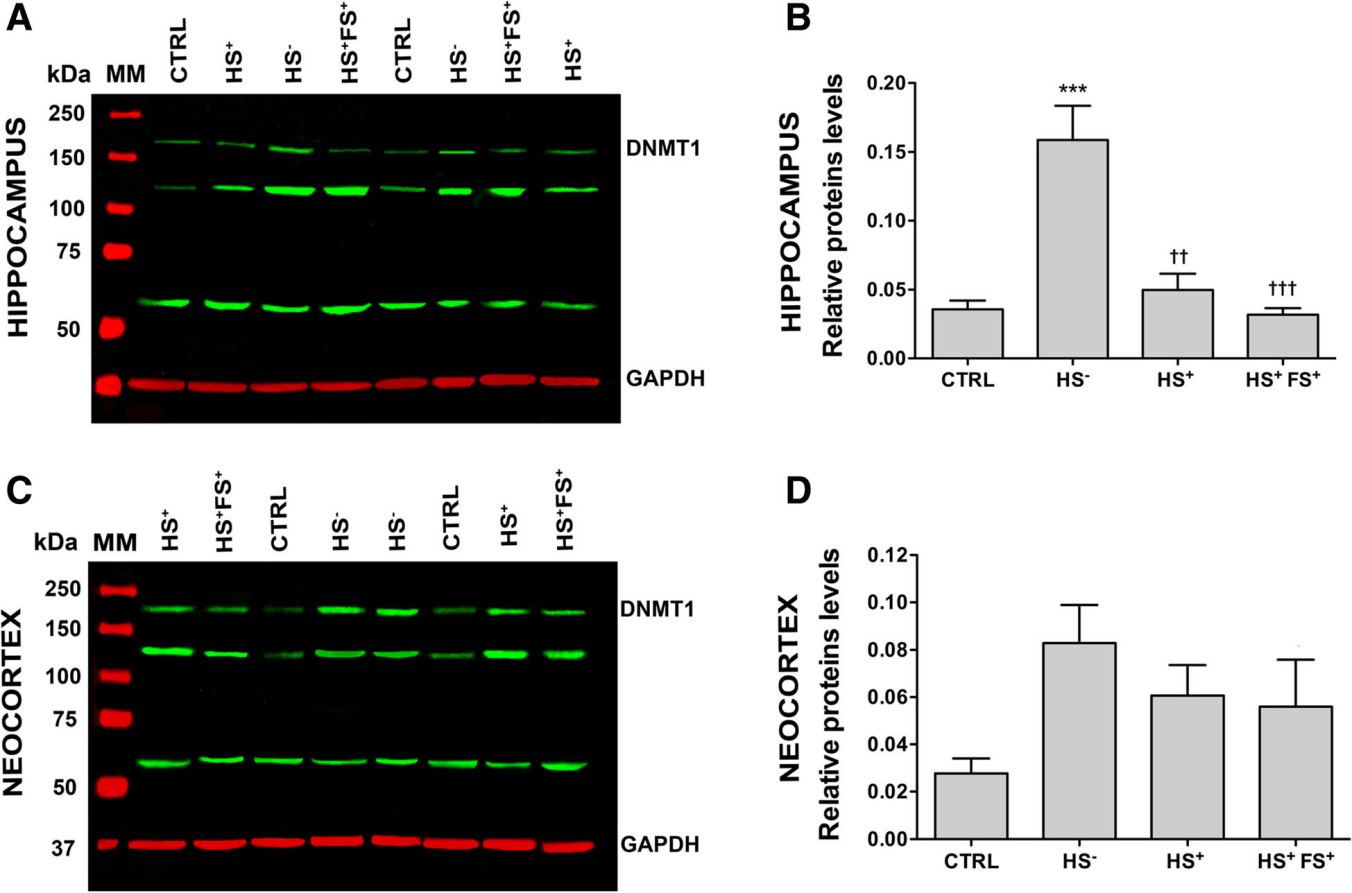
DNMT1 protein expression in the TLE hippocampus (**a**, **b**) and neocortex (**c**, **d**). **a**, **c** Representative Western blot showing DNMT1 (green) in the TLE hippocampus (**a**) and neocortex (**c**) compared to controls. GAPDH (red) was used as a protein loading control. **b**, **d** Graph showing semi-quantification of the Western blots for hippocampus (**b**) and neocortex (**d**). DNMT1 expression was normalized to GAPDH expression. Error bars show SEM. ***p < 0.001 for significant difference to CTRL. ^††^*p* < 0.01 and ^†††^*p* < 0.001 for significant difference to HS^−^ group. MM = molecular weight marker; CTRL = control; HS^−^ = TLE without HS; HS^+^ = TLE with HS; HS^+^FS^+^ = TLE with HS and FS

We first analyzed the expression levels of the two DNMT1 protein isoforms by Western blot using tissue from the hippocampus (Fig. 1a) and temporal neocortex (Fig. 1c) of controls (CTRL) and three TLE patient groups (HS^−^, HS^+^, and HS^+^FS^+^). The HS^+^ group was included in the analysis to control for the possible influence of HS in the HS^+^FS^+^ group. The two isoforms of DNMT1 have a similar molecular weight of 183 kDa and 184 kDa, which urged us to quantify both forms together (here referred to as DNMT1). For normalization, we used GAPDH as a control for protein loading. As shown in Additional file 1A and B, the expression of GAPDH was not different between control and TLE groups in both types of tissues studied. Western blot analysis for DNMT1 revealed the expected band size for DNMT1, as previously described [36], with two extra bands at lower molecular weights (≈ 140 and 60 kDa). In the hippocampus, we found a significantly elevated expression of DNMT1 in HS^−^ subjects when compared to the CTRL group and to the other epileptic groups (Fig. 1b). In the neocortex, DNMT1 expression was higher in all epileptics groups compared to the CTRL group but not at a statistically significant level (Fig. 1d). As expression profiles may be influenced by degradation (in relation to increasing post-mortem intervals), we furthermore tested in mice whether the post-mortem interval influenced the expression levels of the DNMT1. For that purpose, we experimentally varied the post-mortem interval in mice and analyzed the expression of the mouse Dnmt1 in the hippocampus and the neocortex by Western blot. As shown in Additional file 2, Dnmt1 expression did not significantly change up to 24 h post-mortem delay at room temperature neither in the hippocampus (Additional file 2A, B), nor in the temporal neocortex (Additional file 2C, D). Similarly, human DNMT1 expression levels did not correlate with post-mortem interval for human hippocampus and neocortex (Additional file 6, PMI correlation).

### Differential expression of DNMT3a isoforms in the human TLE hippocampus and neocortex

**Fig. 2.**
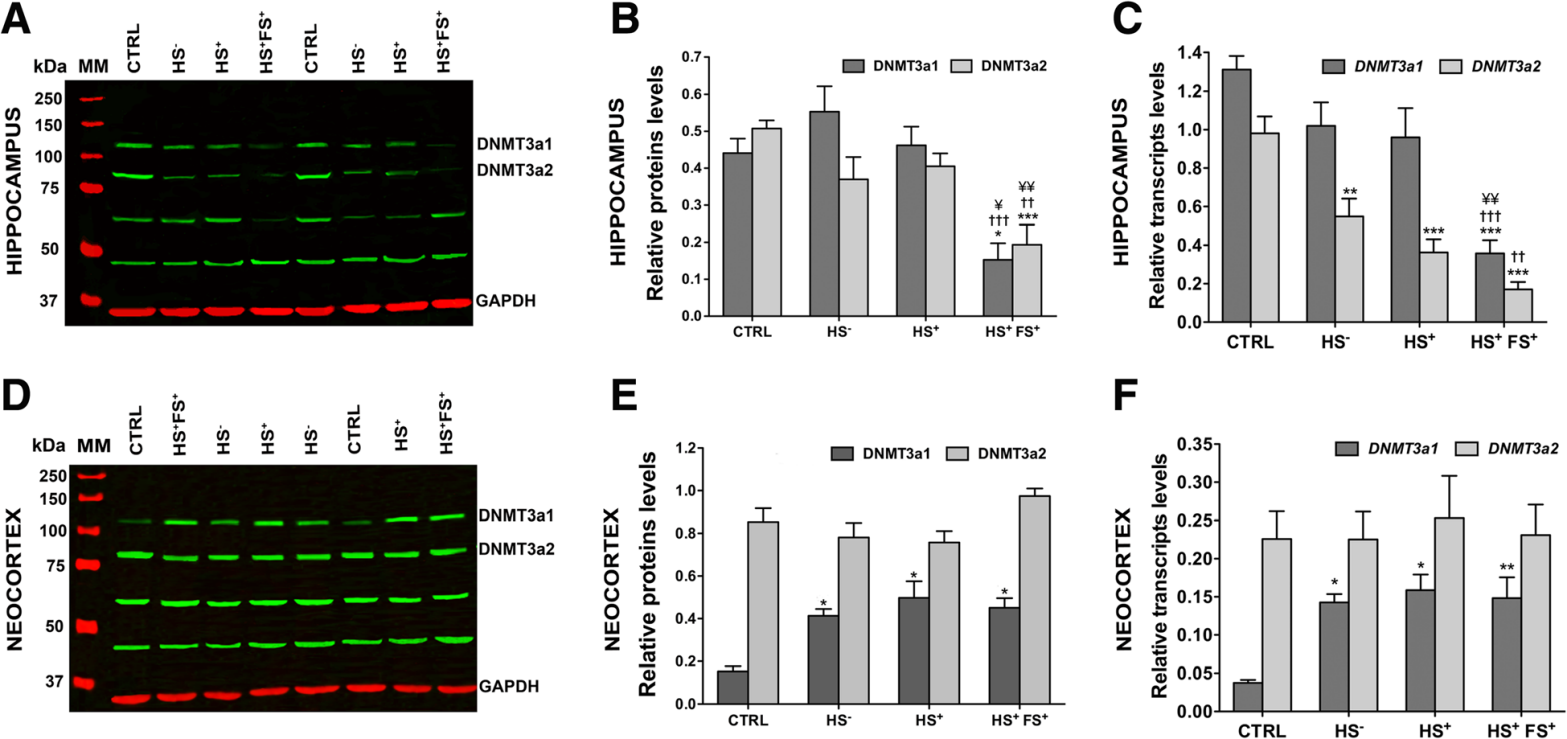
DNMT3a isoforms expression in the TLE hippocampus (**a**–**c**) and neocortex (**d**–**f**). **a**, **d** Representative Western blot showing DNMT3a isoforms (green) in the TLE hippocampus (**a**) and neocortex (**d**) compared to control. GAPDH (red) was used as a protein loading control. **b**, **e** Graph showing semi-quantification of the Western blots for the hippocampus (**b**) and neocortex (**e**). DNMT3a isoforms expression was normalized to GAPDH expression. **c**, **f** Real-time PCR validation of Western blots results for hippocampus (**c**) and neocortex (**f**). Error bars show SEM. *p < 0.05, **p < 0.01, and ***p < 0.001 for significant difference to CTRL. ^††^*p* < 0.01 and ^†††^*p* < 0.001 for significant difference to HS^−^ group. ^¥^*p* < 0.05 and ^¥¥^*p* < 0.01 for significant difference to HS^+^ group. MM = molecular weight marker; CTRL = control; HS^−^ = TLE without HS; HS^+^ = TLE with HS; HS^+^FS^+^ = TLE with HS and FS

As for DNMT1, we analyzed the expression of DNMT3a1 and DNMT3a2 protein isoforms by Western blot in the hippocampus (Fig. 2a) and the temporal neocortex (Fig. 2d) of the same CTRL and TLE groups. GAPDH levels were not significantly different in both tissues studied between CTRL and epilepsy groups (Additional file 1C, D). The blots showed bands of expected size for both isoforms (130 kDa for DNMT3a1 and 81 kDa for DNMT3a2), as previously described [37], with two extra bands at lower molecular weights (≈ 45 and 60 kDa). In the hippocampus, we demonstrated that the expression of DNMT3a1 and DNMT3a2 was statistically significantly diminished in HS^+^FS^+^ subjects compared to CTRL and other TLE groups (Fig. 2b). In order to confirm these findings at the mRNA level, we performed qRT-PCR (Fig. 2c) and obtained the same results for *DNMT3a1.* On the other hand, we found that all TLE groups displayed a significant decrease of *DNMT3a2* transcript expression compared to CTRL (Fig. 2c). Within the TLE groups, patients with FS showed a significant decrease compared to HS^−^ but not to HS^+^ group (Fig. 2c). By contrast, in the neocortex, the protein (Fig. 2e) and mRNA transcript (Fig. 2f) levels of DNMT3a1 were significantly increased for all TLE groups compared to the CTRL. No changes in the expression of the DNMT3a2 protein and transcript were found in the neocortex (Fig. 2e, f).

In order to exclude the possibility that the reduced DNMT3a levels were the results of degradation, the stability of murine Dnmt3a isoform’ protein levels with increasing post-mortem delay was confirmed as described for Dnmt1 (Additional file 3). In addition, human DNMT3 isoform expression did not correlate with post-mortem interval neither within the hippocampus nor in the neocortex (Additional file 6, PMI correlation).

### Decreased expression of DNMT3a2 isoform in the TLE dentate gyrus of the hippocampus

**Fig. 3:**
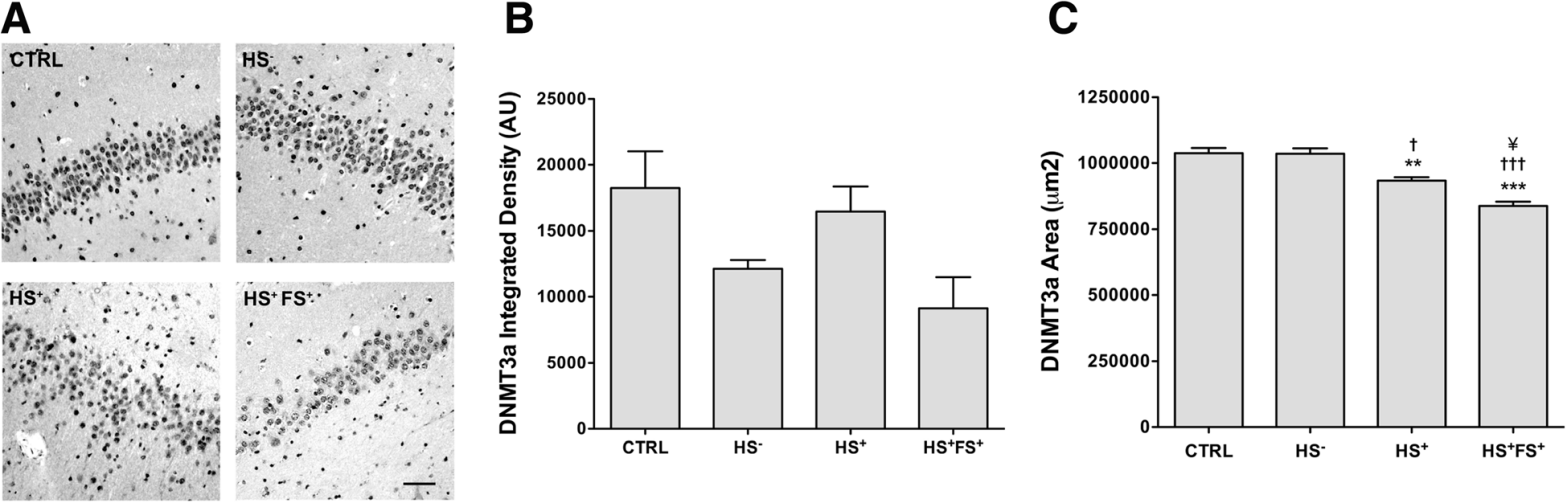
DNMT3a immunoreactivity in the hippocampal DG. **a** Representative pictures of DNMT3a staining in the hippocampal DG of TLE patients and controls. Scale bars represent 100 μm. **b** ImageJ quantification of the integrated density of DNMT3a staining. **c** Stereological quantification of the area expressing DNMT3a. Error bars show SEM in **b** and CE in **c**. **p < 0.01 for significant difference to CTRL. ^†^*p* < 0.05 and ^††^*p* < 0.01 for significant difference to HS^−^ group. ^¥^ *p* < 0.05 for significant difference to HS^+^ group. CTRL = control; HS^−^ = TLE without HS; HS^+^ = TLE with HS; HS^+^FS^+^ = TLE with HS and FS.

In order to further explore whether the decrease in DNMT3a isoforms in TLE hippocampus were specific for different hippocampal sub-regions, we performed immunohistochemistry on the same hippocampal samples using the same antibody used in Western blot experiments. As this antibody recognizes both DNMT3a isoforms, we quantified the expression of both isoforms together (denoted as DNMT3a) and could not specifically analyze DNMT3a1 or DNMT3a2 isoforms using immunohistochemistry. The expression pattern of DNMT3a in the DG is presented in Fig. 3a. The integrated density of DNMT3a immunoreactivity in the DG showed a statistically non-significant decrease in global DNMT3a levels in HS^−^ and HS^+^FS^+^ compared to CTRL, whereas the area containing DNMT3a-positive cells showed a statistically significant decrease in HS^+^ and HS^+^FS^+^ groups compared to CTRL (Fig. 3c).

**Fig. 4:**
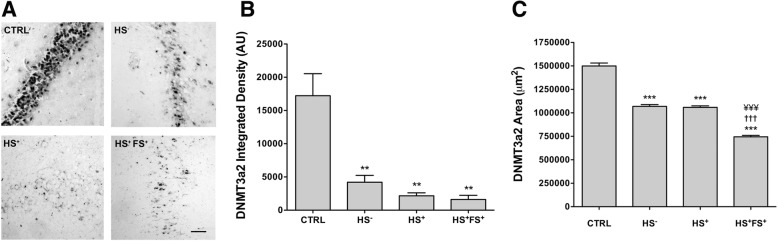
*DNMT3a2* transcript localization in the DG of the hippocampus. **a** Representative pictures of *DNMT3a2* in situ hybridization in the hippocampal DG of TLE patients and controls. Scale bars represent 100 μm. **b** ImageJ quantification of the integrated density of *DNMT3a2* reactivity. **c** Stereological quantification of the area expressing *DNMT3a2*. Error bars show SEM for ImageJ and CE for stereology. **p < 0.01 and ***p < 0.001 for significant difference to CTRL. ^†††^*p* < 0.01 for significant difference to HS^−^. ^¥¥¥^*p* < 0.001 for significant difference to HS^+^. CTRL = control; HS^−^ = TLE without HS; HS^+^ = TLE with HS; HS^+^FS^+^ = TLE with HS and FS.

To explore to which extent the decrease in DNMT3a2 expression level in the hippocampus (Fig. 2) was mediated via the DG, we performed in situ hybridization with *DNMT3a2* mRNA probe (Fig. 4a). Quantifications analysis revealed that all TLE groups displayed a significant decrease of *DNMT3a2* integrated density and the area expressing *DNMT3a2* mRNA compared to CTRL (Fig. 4b, c). Within the TLE groups, patients with FS showed a significant decrease compared to HS^−^ and HS^+^ groups (Fig. 4c).

For these experiments as well, the post-mortem interval did not correlate with the expression of DNMT3a isoforms (Additional file 6, PMI correlation).

### Differences in global 5-mC and 5-hmC levels following TLE

**Fig. 5:**
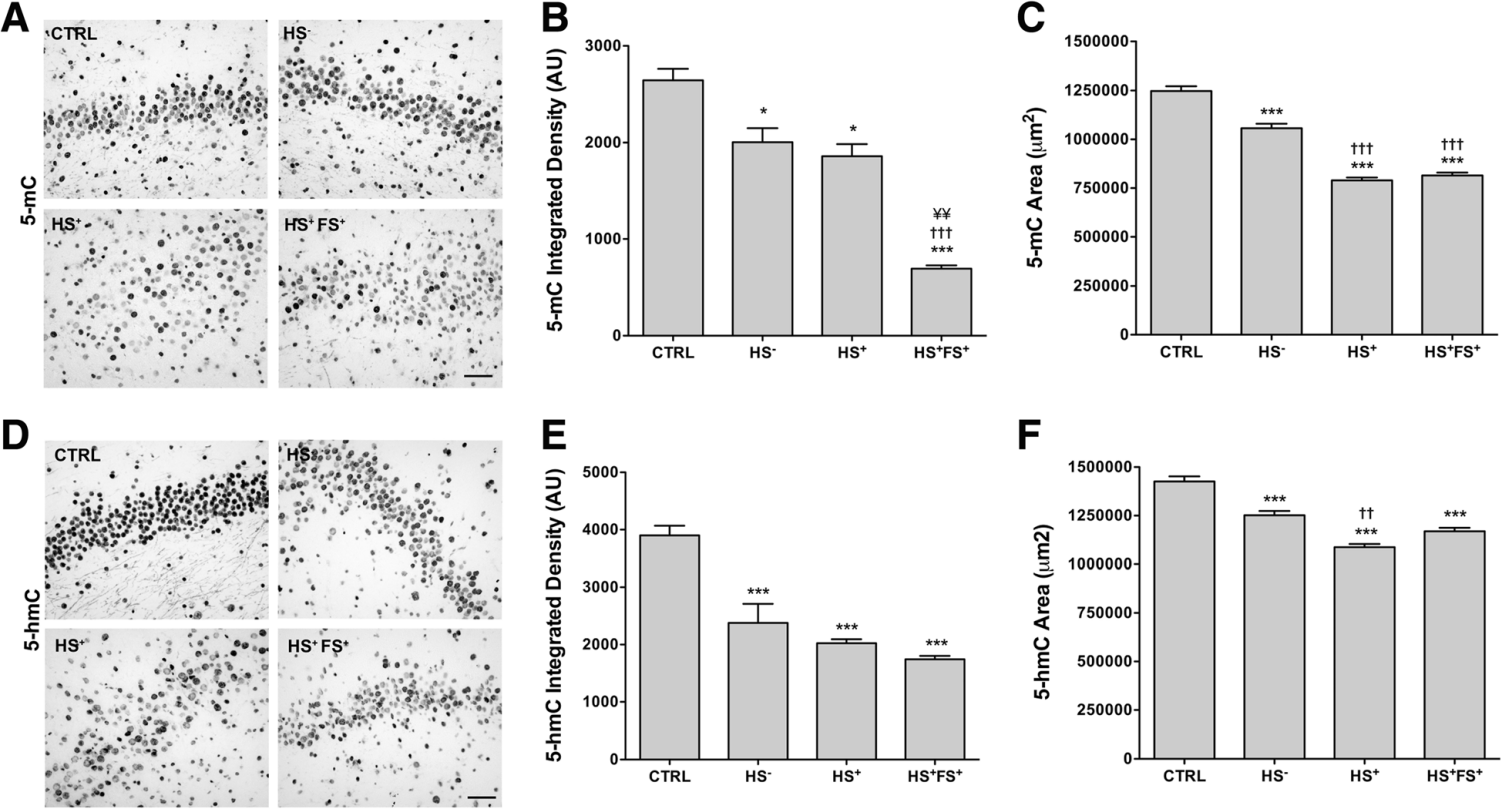
Global 5-mC and 5-hmc patterns in the hippocampal DG. **a**, **d** Representative pictures of 5-mC staining (**a**) and 5-hmc staining (**d**) in the hippocampal DG of TLE patients and controls. Scale bars represent 100 μm. **b**, **e** ImageJ quantification of the integrated density of 5-mc staining (**b**) and 5-hmc staining (**e**). Error bars show SEM. **c**, **f** Stereological quantification of the area expressing 5-mC staining (**c**) and 5-hmc staining (**f**). Error bars show CE. *n* = 3–7 per group. *p < 0.05 and ***p < 0.001 for significant difference to CTRL. ^††^*p* < 0.01 and ^†††^*p* < 0.001 for significant difference to HS^−^ group. ^¥¥^*p* < 0.01 for significant difference to HS^+^. CTRL = control; HS^−^ = TLE without HS; HS^+^ = TLE with HS; HS^+^FS^+^ = TLE with HS and FS

**Fig. 6:**
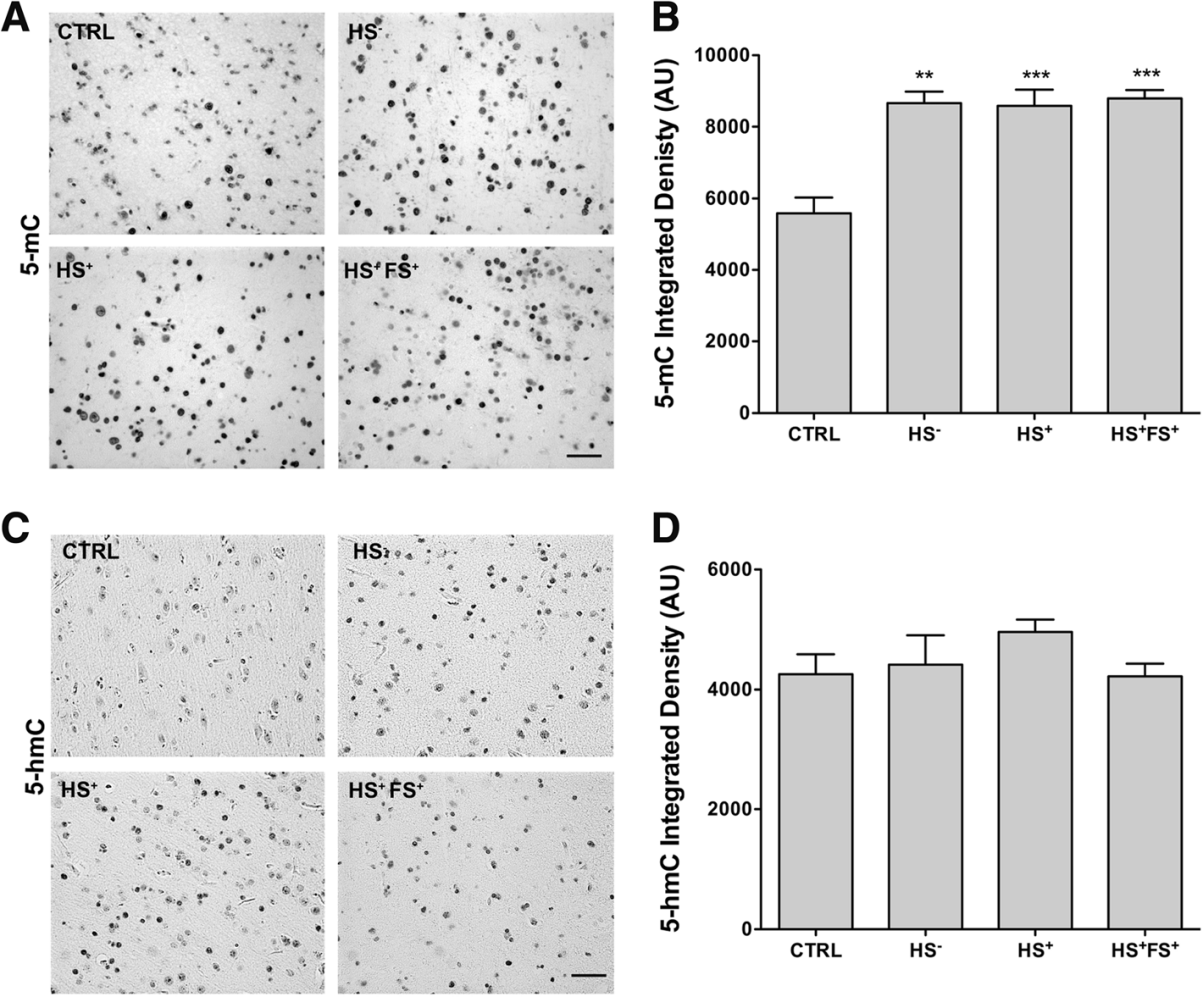
Global 5-mC and 5-hmc patterns in the neocortex. **a**, **c** Representative pictures of 5-mC staining (**a**) and 5-hmc staining (**c**) in the neocortex of TLE patients and controls. Scale bars represent 100 μm. **b**, **d** ImageJ quantification of the integrated density of 5-mc staining (**b**) and 5-hmc staining (**d**) in the neocortex. *n* = 6–8 per group. Error bars show SEM. **p < 0.01 and ***p < 0.001 for significant difference to CTRL. CTRL = control; HS^−^ = TLE without HS; HS^+^ = TLE with HS; HS^+^FS^+^ = TLE with HS and FS

In order to analyze whether the changes in DNMT1 and DNMT3a expression may have impacted on the global levels of DNA methylation and hydroxymethylation, we analyzed 5-mC and 5-hmC levels by immunohistochemistry in the same hippocampal (Fig. 5) and neocortical tissue samples (Fig. 6). Semi-quantitative image analyses revealed a significant decrease in integrated density of 5-mc and 5-hmc in all TLE groups compared to CTRL (Fig. 5b, e). Moreover, 5-mC integrated density was significantly decreased in HS^+^FS^+^ group compared to HS^−^ and HS^+^ ones (Fig. 5b). The area stained for 5-mc and 5-hmc was significantly decreased in the DG of all TLE groups compared to CTRL (Fig. 5c, f). On the contrary, in the neocortex, 5-mC integrated density was significantly increased in all TLE patients compared to CTRL (Fig. 6b), yet no significant difference in 5-hmC was observed (Fig. 6c, d).

Correlation analyses did not reveal any statistically significant association between the levels of 5-mC and 5-hMC and the post-mortem interval (Additional file 6, PMI correlation).

## Discussion

The main aim of the present cross-sectional pilot study was to make a first step in the exploration of the possible involvement of DNA methylation and DNMT isoforms in the pathophysiology of TLE with or without a history of FS. To our knowledge, this is the first to investigate DNMT isoforms and global DNA (hydroxy)methylation profiles in TLE using specimen from surgically treated TLE patients with HS and a history of FS. In the neocortex, we found an increase of DNMT1 and DNMT3a1 expression and global DNA methylation levels for all TLE groups compared to the CTRL. By contrast, we showed that DNMT3a2, global DNA methylation, and hydroxymethylation levels were profoundly lower in the hippocampus, specifically in the DG, for all TLE groups compared to the CTRL, with a more significant decrease amongst the TLE groups with a history of FS. Remarkably, we demonstrated that DNMT3a1 isoform expression was specifically decreased in the hippocampus of TLE patients with a history of FS compared to epileptic groups without FS and CTRL. A summary of the main findings of this study is presented in Table 1. Together, these results provide the first evidence to support the hypothesis that DNA (hydroxy)methylation, DNMT1, and DNMT3a isoforms may be involved in a brain region-specific manner to mediate differential epileptogenesis in TLE with or without a history of FS.

**Table 1:** Summary of the main findings described in this study

Tissue	Variables measured	Methods	Groups comparison	Effect
Neocortex	DNMT1	WB	All TLE groups vs CTRL	↑
Neocortex	DNMT3a1 *DNMT3a1*	WB, qRT-PCR	All TLE groups vs CTRL	↑
Neocortex	5-mC	IHC	All TLE groups vs CTRL	↑
Hippocampus	DNMT1	WB	HS^−^ vs CTRL	↑
Hippocampus	*DNMT3a2*	qRT-PCR, ISH	All TLE groups vs CTRL	↓
Hippocampus	DNMT3a2	WB	HS^+^FS^+^ vs CTRL HS^+^FS^+^ vs HS^−^ HS^+^FS^+^ vs HS^+^	↓
Hippocampus	5-mC, 5hmC	IHC	All TLE groups vs CTRL	↓
Hippocampus	DNMT3a1 *DNMT3a1*	WB, qRT-PCR	HS^+^FS^+^ vs CTRL HS^+^FS^+^ vs HS^−^ HS^+^FS^+^ vs HS^+^	↓

*WB* Western blot, *TLE* temporal lobe epilepsy, *CTRL* control, *qRT-PCR* quantitative reverse transcription polymerase chain reaction, *IHC* immunohistochemistry, *ISH* in situ hybridization, *FS* febrile seizures, *HS* hippocampal sclerosis

Our findings in the neocortex are consistent with a previous study from Zhu et al., showing that DNMT1 and DNMT3a protein expression levels were increased in human neocortex from TLE patients without HS [35]. In line with the results of our study of the hippocampus, previous studies on animal models of status epilepticus have demonstrated a decreased *Dnmt3a* transcript expression and global DNA methylation [31] as well as genome-wide prominent hypomethylation of gene promoters in the hippocampus of cases with epilepsy [29]. In contrast to our results, global DNA methylation patterns were shown to be increased in the hippocampus of a kindling rat model of TLE [33] and in chronic rat epilepsy induced by pilocarpine [30]. Moreover, in the human TLE hippocampus, Kobow et al. found increased methylation of the Reelin promoter associated with granule cell dispersion in the hippocampus [34] and Miller-Delaney and colleagues reported genome-wide hypermethylation in relation to HS [32]. Recently, in a rat model of FS, the expression of Dmnt1 mRNA and protein was found to be increased in the adult hippocampus of animals that experienced FS at post-natal day 10, while DNMT3a expression did not change [38]. The dissimilarities between our results and other studies might be explained by the different experimental setup and time points (acute vs chronic) used, but further studies are required to prove these possibilities. To summarize, our actual work with prior reports concluded that DNA methylation and DNMTs might play a major role in TLE. However, it also demonstrates that DNA methylation as well as DNMT1 and DNMT3a isoform profiles appear to display brain region-specific patterns potentially depending on the etiology of TLE, the stage of the disease development, and ongoing pathology. This hypothesis is further supported by recent work showing dissimilarities in genome-wide methylation changes in animal models and human epilepsies with different etiologies [32, 39, 40]. These results are also in accordance with previous studies showing dissociable roles for DNMT1 and DNMT3a in learning and memory, adult behavior, and synaptic plasticity [25, 26, 41, 42].

A major finding in the present study was that the expression of DNMT3a1 and DNMT3a2 and the levels of global DNA methylation were severely decreased in the hippocampus after FS. Molecular and cellular changes have been well-documented to contribute to FS-induced epileptogenesis. For example, an animal model of FS and human TLE has been associated with increased hippocampal neurogenesis [43, 44]. Moreover, FS have been linked to several comorbidities such as memory impairments [45, 46]. In this regard, one could speculate that FS-induced changes in DNA methylation may transiently or persistently affect gene expression patterns responsible for key phases of the epileptogenesis process and associated comorbidities. In fact, it was demonstrated that DNMT3a play a critical role in adult neurogenesis with DNMT3a deficiency leading to enhanced proliferation of postnatal neuronal stem cells, impaired differentiation to neurons, and increased astrogliogenesis and oligodendrogenesis [47, 48]. In addition, Oliveira and colleagues have reported that DNMT3a2 was an endogenous player in memory formation [27, 28]. Along these lines, cortical infusion of DNMT inhibitors after the establishment of short-term memory has been shown to impair memory consolidation and formation of long-term memory [49]. Therefore, it will be interesting to further investigate (and experimentally test) the possible interpretation of our results, i.e., that decreased expression of DNA methylation, DNMT3a1, and DNMT3a2 could contribute to increased proliferation of newborn cells, astrogliosis, and memory impairment observed in TLE after FS. Clearly, also the cellular mechanisms by which DNMT3a isoforms and DNA methylation contribute to gene expression and cellular changes in FS-induced epileptogenesis need further investigation in rodent models of FS and in human TLE.

There are some limitations of the present study. The first one is that our investigation is a pilot study that was performed using a small sample size. However, the post hoc power calculation demonstrated that the statistically significant results obtained were supported by a power higher than 80%. Another constraint is the lack of age- and gender-matched groups. To circumvent that issue, we included age and gender as confounding factors in the statistical analysis. Moreover, because of the cross-sectional design of the study, we were not able to disentangle whether the observed epigenetic changes are the cause or the consequence of TLE development. Therefore, our main findings should be replicated using a larger cohort matched for age and gender and with a longitudinal design to test for causality.

Another caveat is that post-mortem controls may contain autopsy delay that alters the expression of DNMTs and DNA methylation profiles. The simulation of the post-mortem delay in our murine experiment showed that Dnmt1 and Dnmt3a isoform’ proteins levels were stable until 24 h after death. Regarding DNA (hydroxy)methylation, it has been shown that 5-mC and 5-hmC are stable for more than 48 h post-mortem in human, pig, and mouse brain [50]. Of note, the longer post-mortem interval of our study is 42 h. In addition, correlations analysis between DNMT isoform expressions, 5-mC and 5-hmC levels, and post-mortem interval did not show statistically significant relationship. Together, these indications suggest that the post-mortem delay most likely did not influence the results of our study; however, we could not completely exclude a potential effect.

The use of whole tissue for Western blot and qPCR analyses did not allow to distinguish differences in DNMT isoform expression between hippocampal subfield and cell types (e.g., glia and neurons). Future studies using cell sorting or laser capture microdissected tissues may be able to feature cell type- and subregion-specific epigenetic changes that occur in TLE with or without FS history.

Another important limitation is the measurements of global 5-mC and 5-hmC levels that are unable to specify where in the genome methylation alterations have occurred. A large number of genes might be involved in the development of TLE, each of them is likely to exhibit unique changes in methylation patterns, and depending on the direction of those changes, effects could be washed out in a global assessment. Therefore, future studies investigating gene-specific methylation profiles and gene expression are needed.

Finally, the influence of antiepileptic drugs on DNA methylation status should be taken into consideration. Notably, the effects of antiepileptic drugs on epigenetic factors are not fully understood (for review, see [51]). In this study, long-term treatment of all patients with a combination of several antiepileptic drugs was used before surgery resections and we could not exclude any direct or indirect effect of these drugs on DNMT expression and global (hydroxy)methylation patterns. Future studies using animal models of TLE should be conducted to unravel the potential influence of antiepileptic drugs on DNA methylation.

## Conclusion

In summary, the findings of our descriptive cross-sectional pilot study demonstrated a brain region-specific different pattern of DNMT1 and DNMT3a isoform expression, as well as DNA methylation and hydroxymethylation levels in TLE patients with or without a history of FS. Our results specifically suggest a potential involvement of DNMT3a isoforms in FS-induced epileptogenesis. Therefore, DNMT3a isoforms represent interesting targets for further experimental studies to explore the pathophysiology of TLE with different etiologies and to investigate the causality between FS and TLE. Clearly, the assumed correlation between neuronal hyperexcitability after FS, DNA methylation, and DNMTs needs further study as understanding the exact pathophysiological mechanisms may contribute to the identification of disease biomarkers and new treatment strategies to drug-resistant epilepsy or for preventing FS-induced epileptogenesis.

## Materials and methods

### Human brain tissue

Human hippocampi and neocortices were collected at Maastricht University Medical Center (MUMC) as previously described [52]. In total, 22 TLE patients were included, of which 14 did not show any signs of HS (HS^−^), 10 had the diagnostic of HS (HS^+^), and 13 had HS with a history of FS in childhood (HS^+^FS^+^). Patient demographics are presented in Table 2.

**Table 2:** Clinical characteristics of TLE patients and post-mortem controls

Controls	Gender	Age (years)	PMI (h)	Cause of death		Resection side
C1302	Male	62	20	Sepsis		Right
C1303	Male	66	39	Heart failure		Right
C1304	Male	76	14	Sepsis		Left
C1401	Male	80	22	Heart failure		Right
C1402	Male	66	14	Sepsis		Right
C1403	Male	61	19	Heart failure		Right
C1404	Female	84	13	Abdominal carcinoma		Right
C1405	Female	64	42	COPD		Right
C1406	Female	85	17	Lung carcinoma		Right
C1407	Male	83	12	Heart failure		Right
TLE patients	Gender	Age at surgery (years)	Age of onset (years)	Duration	Pathology	Antiepileptic drugs
E1011	Male	58	53	5	HS^-^	VPA, CBZ, LTG, LEV
E1013	Female	51	24	27	HS^-^	LEV, CBZ, LTG
E1016	Male	37	31	6	HS^-^	LEV, VPA, CBZ, LTG
E1107	Female	60	17	43	HS^-^	LTG, CBZ, LEV, CLB
E1204	Female	40	16	24	HS^-^	PHT, DZP, OXC, CLZ
E1205	Male	20	N/A	N/A	HS^-^	N/A
E1206	Male	47	31	16	HS^-^	CLB, OXC, LEV
E1207	Male	29	20	9	HS^-^	OXC, LEV
E1214	Female	39	30	9	HS^-^	LTG, CBZ
E1302	Female	21	20	1	HS^-^	LEV, LAC
E1306	Female	59	7	52	HS^-^	CBZ, CLB
E1319	Female	41	27	14	HS^-^	LEV, CBZ, CLB
E1402	Female	21	1	20	HS^-^	CLE, LEV
E1404	Male	19	8	8	HS^-^	CBZ, CLZ, LEV
E1009	Female	39	4	35	HS^+^	DZP, CLB, LEV, OXC
E1018	Male	63	2.5	60.5	HS^+^	CBZ, CLB, PHT, CLZ
E1019	Female	43	3	40	HS^+^	LTG, CLZ, DZP, LEV, CLB
E1103	Female	58	54	4	HS^+^	LTG, LEV, OXC, CBZ
E1104	Male	47	3	44	HS^+^	LEV, PHT
E1217	Male	54	17	37	HS^+^	FLU, LTG, MET, ZOP
E1218	Male	28	4	24	HS^+^	CBZ, LTG
E1312	Female	23	19	4	HS^+^	MDZ
E1318	Female	47	11	36	HS^+^	LTG, CLB, TMP, VPA
E1407	Male	55	10	45	HS^+^	MDZ, CBZ, CLB, LEV
E0109	Male	48	30	18	HS^+^FS^+^	LTG, CLB, TPM
E0705	Female	23	6	16	HS^+^FS^+^	LEV, LTG, CLB
E0707	Female	59	N/A	N/A	HS^+^FS^+^	OXC, CLB, LEV
E1014	Female	50	47	3	HS^+^FS^+^	OXC, LEV, CLB
E1017	Female	23	1	22	HS^+^FS^+^	LTG, TPM, CLB, OXC
E1213	Male	60	2	58	HS^+^FS^+^	CBZ, VPA, LTG, DZP
E1219	Male	36	9	27	HS^+^FS^+^	LEV, VPA, OXC
E1221	Female	41	4	37	HS^+^FS^+^	CLZ, CBZ
E1305	Female	20	1	19	HS^+^FS^+^	MDZ, LEV, TMP
E1310	Female	43	28	15	HS^+^FS^+^	LEV, LTG, CLB
E1314	Male	42	8	34	HS^+^FS^+^	LTG, CLB
E1408	Male	33	0	33	HS^+^FS^+^	CBZ, CLB, MDZ
E1416	Female	41	20	21	HS^+^FS^+^	LEV, LTG, CLB

*CBZ* carbamazepine, *CLB* clobazam, *CLZ* clonazepam, *DZP* diazepam, *FLU* flupentixol, *FS* febrile seizures, *HS* hippocampal sclerosis, *LAC* lascosamide, *LEV* levotiracetam, *LTG* lamotrigine, *MDZ* midazolam, *MET* methotrimeprazine, *OXC* oxcarbazepine, *PHT* phenytoin, *PMI* post-mortem interval, *TLE* temporal lobe epilepsy, *TPM* topiramate, *VPA* valproic acid, *ZOP* zopiclone, *N/A* not available

Human control tissue samples (CTRL, *n* = 10) were obtained from autopsy. These control patients died of causes not related to a known neurological disease. Average autopsy delay was 16.7 ± 3.8 h (Table 2).

A detailed description of the samples that were used for the different analyses is presented in the Additional file 4.

The sample size of our study is within the range of other studies that analyzed epigenetic mechanism in human TLE samples [32, 34, 35].

### Western blot

Frozen human hippocampus (containing all hippocampal subregions) and neocortex tissues were homogenized in lysis buffer (50 mM Tris-HCl (pH 7.5), 0.01 M phosphate-buffered saline (PBS), 1% Igepal, 0.1% Triton X-100, 1 mM EDTA, 1 mM EGTA, and protease inhibitors cocktail (Complete, Roche) using a Mini-Beadbeater (GlenMills). Lysates were incubated on ice for 30 min and subsequently clarified by centrifugation at 16,000×*g* for 20 min at 4 °C. Protein concentrations were determined using the DC protein assay (BioRad). Total proteins (50 μg per lane) were separated using a 6 or 8% SDS-PAGE for DNMT1 and DNMT3a, respectively. Each sample was run in triplicate. Proteins were subsequently transferred to a nitrocellulose membrane (Merck Millipore) for 16 h at 90 mA. After 5 min washing in PBS and 1 h blocking in Odyssey Blocking buffer (LI-COR) diluted 1:2 in PBS, the membranes were incubated for 1 h at room temperature with primary antibodies diluted in Odyssey blocking buffer-PBS 1:2 (rabbit anti-DNMT1, sc-20701, Santa Cruz, 1:200; rabbit anti-DNMT3a, sc-20703, Santa Cruz, 1:500; mouse anti-GAPDH, 10R-G109A, Fitzgerald, 1:2,000,000). Membranes were washed once in PBS-0.1% Tween 20 (PBS-T) and twice in PBS for 10 min each and subsequently incubated for 1 h at room temperature with the secondary antibodies diluted in Odyssey Blocking buffer-PBS 1:2 (donkey anti-mouse IRDye 670 and goat anti-rabbit IRDye 800, LI-COR, 1:10,000). After three washings of 10 min (PBS-T, PBS, PBS), immunoreactive protein bands were visualized by an Odyssey Infrared Imaging System (Li-COR).

Immunoblots were analyzed with ImageJ software. Relative pixel intensities were measured, and the background signal was subtracted. DNMT1 and DNMT3a isoform intensities were corrected by their respective GAPDH expression (relative protein levels).

The validations of DNMT1 and DNMT3a antibodies are described by He et al. [36] and Challen et al. [37], respectively.

### qRT-PCR

Total RNA was isolated from frozen human hippocampi and neocortices using the TRIzol reagent (Invitrogen) following the manufacturer’s instructions. RNA was quantified on a NanoDrop 1000 spectrophotometer (NanoDrop Technologies). One microgram of total RNA was converted into first-strand cDNA with oligo(dT) primers using RevertAid First Strand cDNA Synthesis Kit (Thermo Scientific) as described by the manufacturer. qPCR experiments were performed using the Roche 480 LightCycler (Roche Applied Science) with SensiMix SYBR Hi-ROX (Bioline) and specific primers listed in Additional file 5. Minus reverse transcription and non-template controls were used to control for genomic DNA and cross-well contamination, respectively. All samples were run in triplicates, and triplicates discordant in Cq by more than 0.5 cycles were excluded from the analysis. For normalization of mRNA expression, RefFinder was used to find the most stable reference gene among *YWHAZ*, *RPL13a*, *TBP*, and *NSE*. For hippocampus, the relative abundance of mRNAs was standardized with the geometric mean of *TBP/NSE* mRNA, as previously reported [53], while for the neocortex, *RPL13a* and *TBP* were used. Mean normalized expression was calculated by the Pfaffl method that accounts for amplification efficiencies.

### Immunohistochemistry

Formalin-fixed paraffin-embedded tissues were cut at 5 μm on the microtome (Leica). Sections were transferred onto SuperFrost Plus slides (VWR) and dried overnight at 37 °C. Slices were deparaffinized and subsequently subjected to antigen retrieval in 0.01 M citrate buffer pH 6 for 20 min at 95 °C. After cooling down at room temperature for 20 min, sections were washed three times in 1 M tris-buffered saline (TBS) 5 min each. After incubation in TBS containing 1% H_2_O_2_ for 30 min at room temperature, slides were washed three times 5 min in respectively TBS-triton 0.3% (TBS-T), TBS, and TBS-T. Sections were blocked for 30 min at room temperature in TBS-T with 3% normal donkey serum (NDS) before incubation with primary antibodies diluted in TBS-T 0.3% NDS for overnight at 4 °C (rabbit anti-5-hmC, Active Motive, 1:5000; mousse anti-5-mC, Genway Biotech, 1:1000; rabbit anti-DNMT3a, sc-20703, Santa Cruz, 1:500). Slides were washed three times 5 min in TBS-T, TBS, and TBS-T at room temperature. Then, sections were incubated for 1 h at room temperature with secondary antibodies diluted in TBS-T 0.3% NDS (biotinylated donkey anti-rabbit or anti-mouse, Jackson ImmunoResearch, 1:200). After three washing in TBS-T, TBS, and TBS-T (5 min each), slides were incubated for 2 h at room temperature with Vectastain ABC kit (Vector Laboratories) diluted 1:500 in TBS-T. Slides were then washed two times in TBS and one time in Tris-HCl 0.05 M (pH 7.6), and subsequently incubated in 3,3′-Diaminobenzidine (DAB)-Peroxidase Substrate solution (0.05% DAB, 0.015% H_2_O_2,_ Tris-HCl 0.025 M, pH 7.6) for 10 min and washed three times 5 min each in TBS at room temperature. Slides were dehydrated and coverslipped using Pertex mounting medium (HistoLab). Pictures were taken with an Olympus AX70 microscope with bright field illumination coupled to a digital camera (f-view, Olympus) as previously described [52].

### In situ hybridization

Formalin-fixed paraffin-embedded tissues were cut at 5 μm on the microtome (Leica). Sections were transferred onto SuperFrost Plus slides (VWR) and dried overnight at 37 °C. Slices were deparaffinized in xylene, decreasing concentrations in ethanol solutions and PBS at room temperature then treated with 15 μg/ml of Proteinase K for 10 min at 37 °C. Slides were incubated with pre-hybridization solution (USBiological) for 3 h at 60 °C and then hybridized with 40 nM double-DIG-LNA probe (Exiqon) complementary to *DNMT3a2* mRNA overnight at 55 °C. Slides were washed in the following buffers for 10 min each at 55 °C: twice in 5× standard saline sodium citrate (SSC), once in 2× SSC, three times in 5 M tetramethylammonium chloride (TMAC), and twice in 2× SSC. Treatment with 1 μg/ml Proteinase K in 2× SSC was done for 30 min at 55 °C before another series of washing steps of 5 min each: 2× SSC, 1× SSC, and 0.2× SSC (all at 55 °C), two times in 0.2× SSC at 37 °C and once in 0.2× SSC at room temperature. Slides were then rinsed twice with PBS for 10 min at room temperature, blocked for 1 h in blocking solution (USBiological), and incubated with anti-DIG alkaline phosphatase-conjugated antibody overnight at 4 °C (USBiological). Tissues were washed with Alkaline Phosphatase buffer twice for 5 min at room temperature and subsequently incubated in nitroblue tetrazolium/5-bromo-4-chloro-3indolyl phosphate (NBT/BCIP) color substrate at room temperature in the dark for 72 h. The slides were rinsed with water, coverslipped with aqua mount (Lerner Laboratory). Positive controls and no-probe controls were included for each hybridization procedure.

### Microscopy and image analysis

For intensity measurements, images were taken with the × 10 objective using a bright-field filter for each staining. All images were taken using identical camera, microscope lens, and light settings. One hippocampal/neocortex section was used per each staining, where images were taken from five sites in the DG per subject. All images for signal intensity analysis were taken with Olympus AX70 Grey value microscope. ImageJ version 1.51j8 was used to calculate the integrated density of the staining. The threshold baseline was calculated by averaging the thresholds of three randomly selected DG pictures (beginning, middle, and end) from all the sections, both control and TLE slides. The setting was as follows: image was inverted, type was 32 bits, background pixel was set to NaN, and set measurements were area, mean gray value, standard deviation, min and max gray value, and integrated density.

For stereology measurements, live image of DNMT3a, 5-mC, and 5-hmC stained slides were observed under bright field using the stereology microscope Olympus BX51. The configuration of the stereology microscope consists of Olympus BX51, LEP MAC5000, and MBF CX9000 camera. The DG expressing the desired probe was manually drawn using StereoInvestigator program at × 10 magnification. The software automatically quantified the area (μm^2^) with its corresponding coefficient of error (CE).

### Murin post-mortem interval

NMRI mice were killed by cervical dislocation. Hippocampus and temporal neocortex tissues were collected either immediately after death or following a 1, 8, 16, or 24 h delay at room temperature. Tissue samples were dissected in PBS at 4 °C, immediately frozen on dry ice and stored at − 80 °C. Mice were provided by the GIGA-neuroscience department of the University of Liège. They were used for another study involving embryonic research. They were treated according to the guidelines of Belgian Ministry of Agriculture in agreement with European Community Laboratory Animal Care and Use Regulations.

### Statistics

Statistical analyses were carried out using RStudio (version 1.2.1335), and graphs were built using GraphPad Prism software (version 5.0a). Values are either presented as mean and standard error of the mean or presented as mean and coefficient of error (Stereology). The normal distribution of the data was proved using the Shapiro-Wilk test. The one-way ANOVA followed by Bonferroni post hoc was used to test for multiple comparisons between groups. Age and gender have been used as confounding factors in the analyses. Correlation analyses were done by calculating the Pearson’s correlation coefficient. Differences between the groups were considered significant for *p* ≤ 0.05. During experiments, the experimenter was blinded to the experimental condition to prevent biased assessment. Cohen’s *d* effect size was used to perform two-tail post hoc power analysis using G*Power software (version 3.1.9.4) [54]. A summary of the statistical analysis is provided in Additional file 6.

## Additional files

Additional file 1: GAPDH expression levels in Western blots analysis. Quantification of the expression levels of GAPDH in the Western blots analyzing DNMT1 (A, B) and DNMT3a (C, D) in the hippocampus and the neocortex. (PDF 1126 kb)

Additional file 2: Effect of post-mortem interval on murine Dnmt1 expression. (A, C) Representative Western blots of Dnmt1 (green) in the mouse hippocampus (A) and neocortex (C) extracted immediately (0 h) or after 1, 8, 16, or 24 h delay at room temperature. Gapdh (red) was used as a protein loading control. (B, D) Graph showing semi-quantification of the Western blots (*n* = 2 per time point) for the hippocampus (B) and neocortex (D). Data are presented as relative expression normalized to Gapdh. Error bars show SEM. MM: molecular weight marker. (PDF 3164 kb)

Additional file 3: Effect of post-mortem interval on murine Dnmt3a isoforms expression. (A, C) Representative Western blots of the various Dnmt3a isoforms (green) in the mouse hippocampus (A) and neocortex (C) extracted immediately (0 h) or after 1, 8, 16 or 24 h delay at room temperature. Gapdh (red) was used as a protein loading control. (B, D) Graph showing semi-quantification of the Western blots (*n* = 2 per time point) for the hippocampus (B) and neocortex (D). Data are presented as relative expression normalized to Gapdh. Error bars show SEM. MM: molecular weight marker. (PDF 2061 kb)

Additional file 4: Detailed description of the clinical characteristics of TLE patients and post-mortem controls. (XLSX 43 kb)

Additional file 5: Sequences of the qPCR primers. (XLSX 35 kb)

Additional file 6: Summary of the statistics. (XLSX 37 kb)

## Data Availability

The datasets used and analyzed during the current study are available from the corresponding author on reasonable request. Personal sensitive data from patients will not be made available for ethical reasons.

## Abbreviations

5-hmC: 5-Hydroxymethylcytosine
5-mC: 5-Methylcytosine
CA: Cornu ammonis
CTRL: Control
DG: Dentate gyrus
DNMT: Methyltransferase
FS: Febrile seizures
HS: Hippocampal sclerosis
TLE: Temporal lobe epilepsy
