# Supplementary figures and images for: DNA methyltransferase isoforms expression in the temporal lobe of epilepsy patients with a history of febrile seizures
Source: Clin Epigenetics. 2019 Aug 19;11:118. doi: 10.1186/s13148-019-0721-2 (PMC6701147; doi:10.1186/s13148-019-0721-2)

# Additional file 1

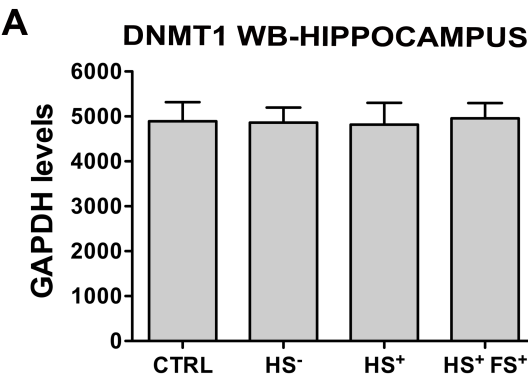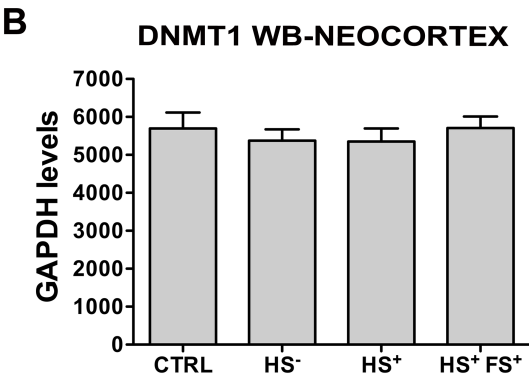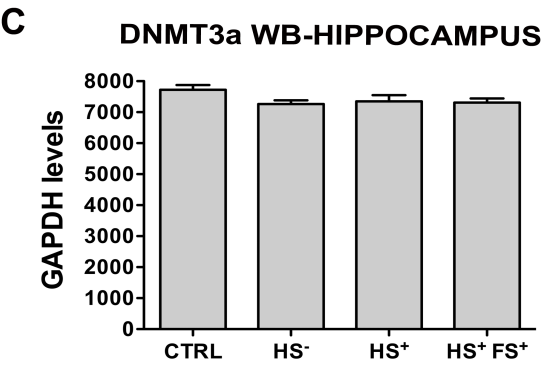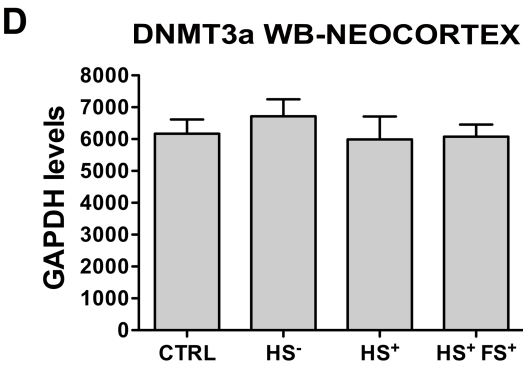

Supplement: Supplementary file 1 — GAPDH expression levels in Western blots analysis. Quantification of the expression levels of GAPDH in the Western blots analyzing DNMT1 (A, B) and DNMT3a (C, D) in the hippocampus and the neocortex. (PDF 1126 kb) [file 13148_2019_721_MOESM1_ESM.pdf]

# Additional file 2

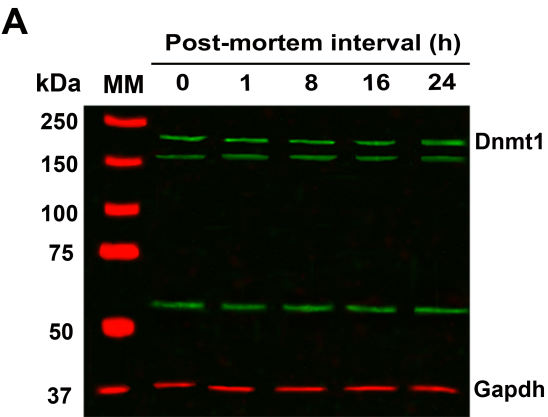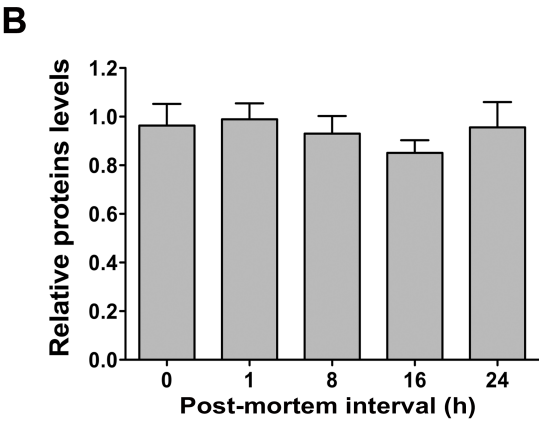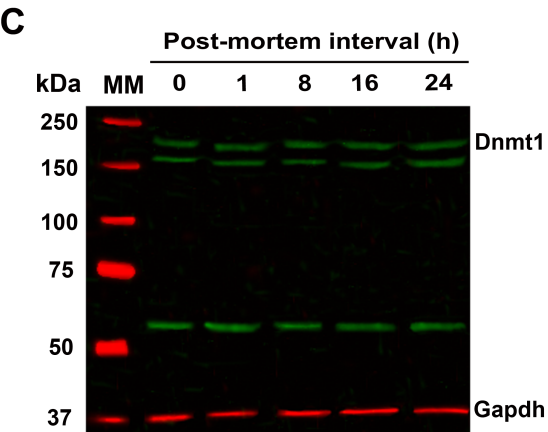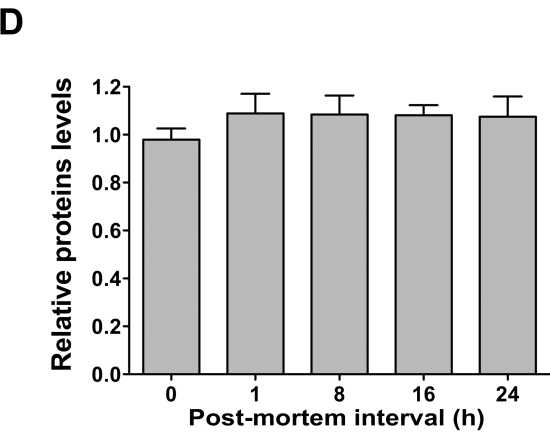

Supplement: Supplementary file 2 — Effect of post-mortem interval on murine Dnmt1 expression. (A, C) Representative Western blots of Dnmt1 (green) in the mouse hippocampus (A) and neocortex (C) extracted immediately (0 h) or after 1, 8, 16, or 24 h delay at room temperature. Gapdh (red) was used as a protein loading control. (B, D) Graph showing semi-quantification of the Western blots (n = 2 per time point) for the hippocampus (B) and neocortex (D). Data are presented as relative expression normalized to Gapdh. Error bars show SEM. MM: molecular weight marker. (PDF 3164 kb) [file 13148_2019_721_MOESM2_ESM.pdf]

Additional file 3

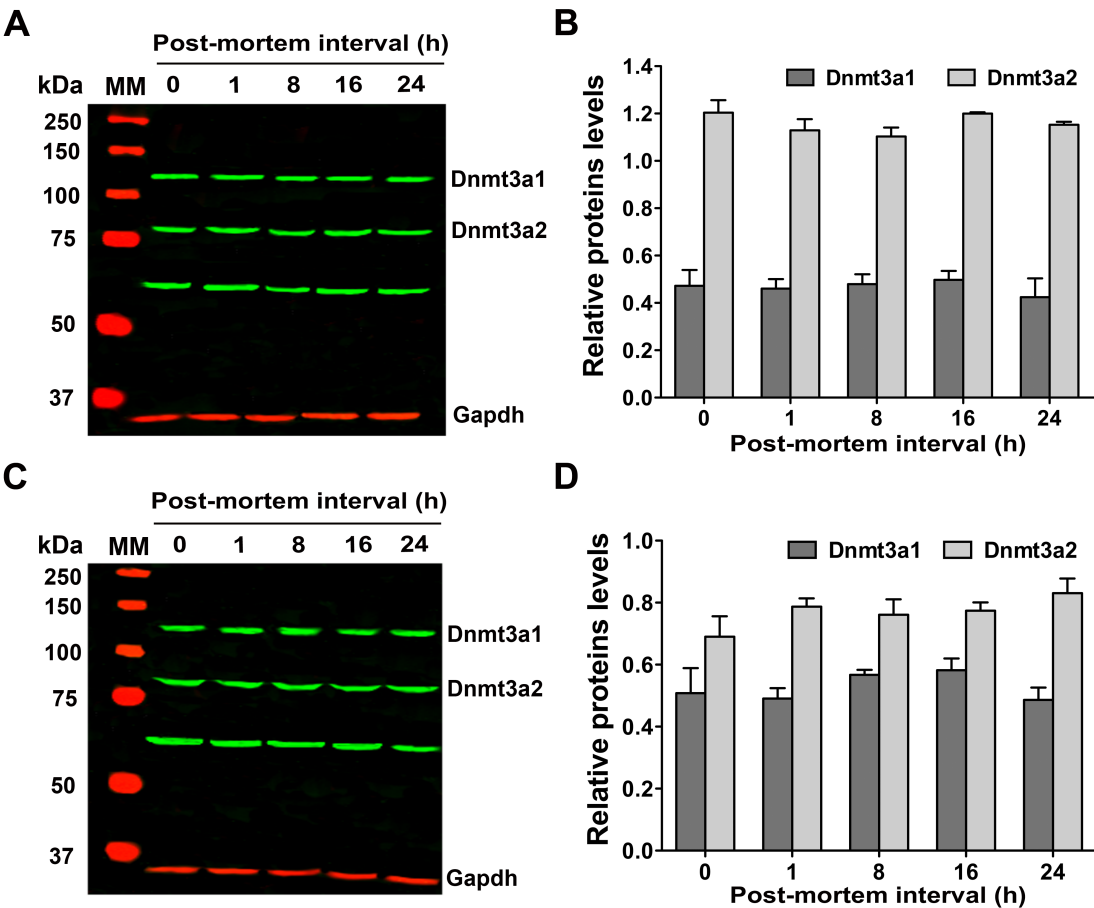

Supplement: Supplementary file 3 — Effect of post-mortem interval on murine Dnmt3a isoforms expression. (A, C) Representative Western blots of the various Dnmt3a isoforms (green) in the mouse hippocampus (A) and neocortex (C) extracted immediately (0 h) or after 1, 8, 16 or 24 h delay at room temperature. Gapdh (red) was used as a protein loading control. (B, D) Graph showing semi-quantification of the Western blots (n = 2 per time point) for the hippocampus (B) and neocortex (D). Data are presented as relative expression normalized to Gapdh. Error bars show SEM. MM: molecular weight marker. (PDF 2061 kb) [file 13148_2019_721_MOESM3_ESM.pdf]
